# Relationship between Health Locus of Control and Risky Sexual Behaviors among Nigerian Adolescents

**DOI:** 10.4172/2155-6113.1000471

**Published:** 2015-06-10

**Authors:** Jennifer Pharr, Victor Enejoh, Bertille Octavie Mavegam, Ayodotun Olutola, Haruna Karick, Echezona E Ezeanolue

**Affiliations:** 1 Department of Environmental and Occupational Health, University of Nevada, Las Vegas, USA; 2 HealthySunrise Foundation, USA; 3 Centre for Clinical Care and Clinical Research, Nigeria; 4 Department of Psychology, University of Jos, Nigeria; 5 Department of Pediatrics, University of Nevada School of Medicine, USA

**Keywords:** Health locus of control, Risky sexual behaviors, Adolescents, Nigeria

## Abstract

HIV/AIDS knowledge has been rated as the most important factor for HIV prevention. However, studies have also shown that knowledge alone does not always translate into reduced risky sexual behavior (RSB). Health locus of control (HLC) categorized as perceived control over health status (internal locus of control) or attribution of health status to chance or fate (external health locus of control) is a psychological construct that has been shown to impact health outcomes including RSB. This study thus investigated the relationship between HLC and RSB among Nigerian adolescents.

A cross-sectional survey design was employed among 361 adolescents from nine senior secondary schools selected through stratified random sampling from Jos, Plateau State Nigeria. Data were collected between August and October of 2008. Health Locus of Control Scale was used to categorize individuals into having either an internal or external HLC. RSB was assessed using the Brief HIV Screener (BHS). Descriptive statistics were computed and Mann-Whitney U test was used to determine differences in BHS scores by HLC categories. Odds ratios and adjusted odds ratios were calculated for individual BHS question responses based on HLC.

Participants were 169 males (46.8%) and 192 females (53.2%) with a mean age of 16.9. When grouped into HLC categories, 141 were internal and 220 were external. The mean score on the BHS showed statistically significant difference based on HLC (p=0.01). Odds for using a condom during sexual intercourse were higher for adolescents with an internal HLC while adolescents with an external HLC had significantly higher RSB scores. Prevention programs targeted at adolescents should also aim to internalize their HLC.

## Introduction

Globally, an estimated 35 million people were living with HIV in 2013 [1]. Adolescents and young adults aged 15 and 24 accounted for 4 million while 3.2 million individuals living with HIV were children younger than 15 years [1]. Nigeria has the second highest burden of HIV and the highest number of HIV related deaths [1]. The prevalence of HIV among adolescents aged 15 to 19 in Nigeria is 3.5% [1]. Additionally, Nigeria has low rates of antiretroviral (ARV) therapy with an estimated 21% of HIV positive people over the age of 15 on ARV therapy and only 12% of children under the age of 14 on ARV therapy [1]. Because of the high prevalence of HIV, high number of HIV related deaths and low ARV therapy in Nigeria, it is important to identify preventive measures to arrest the spread of HIV, particularly among adolescents. Knowledge about transmission and prevention of HIV/AIDS has been rated as the most important foundational factor for prevention [2]. However, studies have also shown that this knowledge does not always translate into reduced risky sexual behavior (RSB) [3-5]. Other factors that contribute to RSB need to be explored. Health locus of control (HLC); whether perceived control over health status (internal locus of control) or attribution of health status to chance or fate (external health locus of control) is a psychological construct that has been shown to impact health outcomes including RSB [6-8].

## Locus of control

The construct of locus of control was developed by Julian Rotter, a psychologist in the 1960s. The concept was originally developed within the framework of Rotter’s Social Learning Theory [9]. Rotter defined locus of control as the perception one has of the control he or she has over the events that occur in his/her life with this trait having an internal or external dimension [9]. Internal control refers to the perception of events, either positive or negative, being a consequence of one’s own actions and thereby under personal control. External control refers to the perception of positive or negative events as being unrelated to one’s own behavior and therefore beyond personal control [10]. The concept of locus of control was extended by Wallston, Wallston, Kaplan and Maides to include health [11]. HLC is the degree to which individuals believe that their health is controlled by internal or external factors [11]. The construct of HLC assumes that people with an internal HLC are more likely to engage in positive and protective health behaviors and take control of their own health. People with an external HLC view their health as out of their control and thus engage in less positive and protective health behaviors while believing that others are responsible for their health [11,12].

HLC in conjunction with adolescents’ risky sexual behavior (RSB) has not been studied extensively and most studies conducted have been limited to the United States and Western Europe. In a study of HLC and RSB of African American adolescents, St. Lawrence found that adolescents with an internal locus of control were more likely to use condoms than adolescents with an external locus of control [6]. In a similar study of adolescents in England, Mendolia and Walker found that adolescents with an external locus of control had a 15 to 16% higher risk of a past unprotected sexual intercourse and were younger than sixteen years at first sexual intercourse than adolescents with internal locus of control [7]. Additionally, Burnett, Sabato, and Smith evaluated the influence of locus of control on college adolescents’ sexual behavior and substance use [8]. They found that university adolescents in the United States with an external locus of control were more likely to adopt unsafe sexual behavior.

No studies were found that examined the role or influence of HLC on adolescents’ RSB in Nigeria. Because of the high prevalence on HIV among adolescents in Nigeria, the purpose of this study was to investigate the relationship between HLC and RSB in Nigerian adolescents.

## Methods

### Setting and sample

A cross sectional design was employed for this study. Nine secondary schools in Jos Plateau state in Nigeria were selected through stratified random sampling. Two classes were then randomly selected at each of the nine schools, one class from senior secondary school 1 and one from senior secondary school 2 (grades 10 and 11). Of the nine schools that were randomly selected, five were state-owned public schools while four (4) were privately (missionary/church) owned. One school enrolled only male adolescents and another one only enrolled female adolescents. The other seven schools were co-educational. Prior to conducting this study, permissions and consent were obtained from the State Ministry of Education, school principals and adolescents. The School Board and the University of Jos, Department of Psychology ethics committee provided ethical approval for this study. Data collection occurred between August and October of 2008.

Participation by the adolescents was voluntary and questionnaires were completed anonymously. Participants were administered the Health Locus of Control Scale (HLC), the Brief HIV Screen (BHS) questionnaire and were also asked to provide some basic demographic information (age and gender). This study was conducted by the researchers with the assistance of a teacher. A school period was taken out of a school day for the adolescents to complete the questionnaires. This was done to avoid disruption in the school routine. A total of 450 questionnaires were administered; however, only 361 were usable for this study. Fifty-six were returned blank, 30 not properly completed while 13 were not returned.

### Survey instruments

The Health Locus of Control Scale (HLC) was developed by Wallston, Wallston, Kaplan and Maides to measure an individual’s attitude regarding perceived control over personal health, with individuals having either an internal or an external HLC [11,13]. The questionnaire contains 11 items on a 6 (six) point likert scale anchored by strongly disagree at one end and strongly agree at the other end with higher scores indicating external HLC and lower scores internal HLC. Those scoring at or above the median are labelled “health externals” and those scoring below are labelled “health internals”. The instrument has test-retest reliability correlation with Rotters’ internal-external scale (I – E), a well-established scale to measure generalized internality/externality locus of control [11,13].

The Brief HIV Screener (BHS) was developed by Gerbert, Bronstone, McPhee, Pantila and Allerton to assess HIV risk, sexual risk and intravenous drug use [14]. It is a 16 item questionnaire and can be self-administered. The four additional items adapted from Peltzers and Promtussananon were included because these items were considered to reflect local and cultural knowledge about HIV and sexual behaviors of adolescents in Nigeria [3]. This was confirmed in a pre-test pilot with twenty adolescents which had a Cronbach’s alpha of .65 [3].

### Data analysis

Data were analyzed using SPSS 21. Descriptive statistics were computed. Because the dependent variable of BHS score was not normally distributed, Mann-Whitney U tests were used to determine differences in BHS scores by HLC categories and gender. For individual dichotomous variables on the BHS questionnaire (i.e. sexually active – yes/no, used a condom at first sexual intercourse – yes/no), chi square and odds ratios (ORs) were calculated comparing internal to external HLC. Adjusted odds ratios (AORs) were also calculated to control for age and gender. P-value for statistical significance was set at < 0.05.

## Results

### Descriptive statistics

Participants were 169 male (46.8%) and 192 female (53.2%). The mean age was 16.9 with a range of 12-24. The mean HLC score was 38.5 with a standard deviation (SD) of 5.6. HLC score was not significantly related to age or gender. When grouped into categories, 141 were internal and 220 were external. The mean score on the BHS was 1.58 with a significant difference based on gender (p<0.01) and age (p<0.01). Males had a higher BHS score (mean = 2.02) than females (mean = 1.19) and BHS score increased with age.

### Risky sexual behavior

Responses on the BHS for RSB items are presented in Table 1. Sixty-two percent of adolescents indicated they had not used a condom during their first intercourse while 56% reported not using a condom during their last intercourse. Additionally, 40% reported always using a condom during intercourse. One third of the adolescents (31.5%) indicated that they had sex with someone much older than them while 31% of the adolescents indicated that they had two or more sexual partners in the past ten years.

Twenty-six (24.1%) of the sexually active adolescents reported having experienced anal sex. Twenty-two percent admitted to having had a sexually transmitted disease, while 25.9% report that one of their sex partners had a sexually transmitted disease. Twenty-seven percent reported that they have had sex to receive gifts while 21.3% had given gifts to receive sex. Thirteen percent admitted injecting street drugs while 22.2% believed that one of their partners had injected street drugs. Thirty-one percent reported that one of their sexual partners had been a man who has sex with other men.

### Health locus of control and risky sexual behavior

The mean BHS score (1.1) was lower for internal HLC adolescents compared to external HLC adolescents’ BHS score (1.9). Mann-Whitney U test revealed a significant difference in BHS score based on HLC (p=0.01).

### Odds ratios

For condom use questions of the BHS, adolescents with an internal HLC were more likely to report their use. Adolescents with an internal HLC were 2.3 times more likely to report having used a condom at their first sexual intercourse, were 2.8 times more likely to report having used a condom the last time they had sex, and were 4.2 times more likely to report always using a condom when having sex. For other BHS questions, there was not a statistically significant difference between adolescents with an internal versus external HLC (Table 2). Because age and gender were significantly related to the total BHS score, we calculated AORs controlling for age and gender. AORs findings remained the same as unadjusted ORs. Adolescents with an internal HLC were significantly more likely to use a condom while age and gender were not significantly related to this behavior.

## Discussion

Adolescents who attributed their health status to chance or fate (external HLC) had significantly higher RSB scores compared to those with an internal locus of control. Additionally, adolescents with an internal HLC were significantly more likely to report using a condom during sexual intercourse. Although RSB was related to gender and age with males engaging in more RSB and RSB increasing with age, gender and age were not significantly related to condom use. These findings support previous research examining HLC and RSB among adolescents in the US and Western Europe, particularly condom usage [6-8].

In this study, adolescents with an internal health locus of control had lower mean scores for RSB than those with an external locus of control. The implication of this is that when young people are made to believe they control what happens to them in terms of health, they will engage in less risky behavior. This can be explained by examining the relationship between locus of control and health. Internal locus of control has been found to be a meditating factor for taking actions to prevent health problems [13,15]. Wallston and Wallston assert that people with an internal locus of control have a sense of responsibility for their own health [13]. While those with an external locus of control believe that fate, luck, random events and chance determines their health status. Thus external loci of control people are less careful and cautious about their health while internals are cautious and will take deliberate steps to protect their health [12,16].

This hypothesis is supported by the finding in this study that adolescents with an internal locus of control had significantly higher odds of using a condom than those with an external locus of control. Condom use was not related to age or gender; it was only related to HLC. Internal HLC individuals realize that their choices impact their health and are more likely to make healthy choices, such as use a condom, when compared to external individuals [7,16]. However, we did not find a significant relationship between other RSB and HLC. Adolescence is a time of physical, psychological, emotional and social transition [17]. The onset of puberty is linked with the initiation of sexual activity and risk-taking is considered to be a normal part of adolescence [17]. Adolescents in this study were equally likely to be sexually active, to have had more than two partners in the past ten years or to have had sexual intercourse with an older partner, regardless of their HLC. These findings may be related to the transitional time period of adolescence in general. However, we did find that adolescents with an internal HLC were more likely to practice safer sex by using a condom. This finding has implications for HIV transmission.

While research has shown that an internal locus of control is associated with positive outcomes for adolescents (i.e. improved general wellbeing, academic success, reducing health risk behaviors), the antecedents of an internal locus of control are not well known [18,19]. Ahlin and Antunes examined the influence of family management strategies, peer interactions, neighborhood context, and individual-level characteristics on internal locus of control in adolescents [19]. While they found that most of the factors influenced internal locus of control, family management strategies was the most influential. Adolescents’ whose parents supervised time spent at home were more likely to have an internal locus of control while adolescents’ whose parents used harsh discipline were more likely to have an external locus of control. This finding may imply that comprehensive HIV prevention programs which attempt to internalize locus of control may need to include a parent education component. Serin, Serin and Sahin suggested training in personal development programs can change university adolescents’ locus of control from external to internal [20]. However, methods of improving internal health locus of control or changing external locus of control to an internal one among adolescents have not been broadly studied. The limited amount of research regarding mechanisms to internalize locus of control highlight the need for additional research in this area.

This study does have limitations. Because it was cross sectional, causation cannot be determined. This study was conducted in Jos Plateau state in Nigeria with adolescents enrolled in nine secondary schools. Results may not be generalizable to other states in Nigeria or to adolescents not enrolled in school.

## Conclusion

This study identifies HLC as a contributing factor to RSB and raises important public health implications. Participants who attribute their health status to chance or fate (external HLC) had significantly higher RSB scores and were less likely to wear a condom when compared to those with an internal HLC. Prevention programs targeted to adolescents should also aim to internalize their HLC. However, more research is needed to identify effective mechanisms to internalize locus of control among adolescents.

## Figures and Tables

**Table 1:** Risky Sexual Behaviours as Measured with the Brief HIV Screener.

	Questions	Yes N, %	No N, %	Don’t know N, %
1	Have you ever had sexual intercourse?	108 (29.9)	253 (69.1)	
2	Did you use a condom at the first sexual intercourse?	41 (38)	67 (62)	
3	The last time you had sexual intercourse; did you or your partner use a condom?	47 (43.5)	61 (56.5)	
4	Did You Ever Have Sex With Someone Much Older Than You (above 30)	34 (31.5)	74 (68.5)	
5	Have you had 2 or more sexual partners in the past 10yrs?	34 (31.5)	74 (68.5)	
6	Have you had anal sex (a man puts his penis into the anus of another person) with any of your sexual partners during the past ten years?	26 (24.1)	82 (75.9)	
7	Do you always use a condom during sexual intercourse (including anal sex)	40 (37)	68 (63)	
8	Have you ever had a sexually transmitted disease such as gonorrhoea, syphilis, chlamydia, genital warts, or genital herpes?	24 (22.2)	84 (77.8)	
9	Have you ever given money, gifts or drugs to anyone to have sex with you?	23 (21.3)	85 (78.7)	
10	Have you ever had sex with someone so that they would give you money, gifts or drugs?	29 (26.9)	79 (73.1)	
11	Have you ever injected street drugs, steroids or vitamins with a needle?	14 (13)	94 (87)	
12	Have any of your sexual partners in the past ten years ever injected street drugs, steroids or vitamins with a needle?	24 (22.2)	69 (63.9)	15 (13.9)
13	Have any of your sexual partners in the past 10years been men who have sex with other men?	34 (31.5)	60 (55.6)	14 (13)
14	Have any of your sexual partners in the past ten years ever had a sexually transmitted disease, such as gonorrhoea, syphilis, chlamydia, genital warts or genital herpes?	28 (25.9)	67 (62)	13 (12)

**Table 2:** Odds Ratios and Chi Square for Risky Sexual Behaviors Based on Health Locus of Control (HLC) – Internal HLC as Reference.

Response to Question	Odds Ratios OR (95% CI)		Chi Square p value
Sexually active	1.43	(.91-2.26)	0.08
Used a condom at the first sexual intercourse	2.29	(1.04-5.06)	0.03
Used a condom at last sexual intercourse	2.77	(1.26-6.08)	0.01
Had sex with someone much older (above 30)	1.15	(.51-2.62)	0.45
Had 2 or more sexual partners in the past 10yrs	1.69	(.73-3.91)	0.15
Had anal sex with any sexual partners during the past ten years?	1.63	(.65-4.08)	0.21
Always use a condom during sexual intercourse (including anal sex)	4.16	(1.82-9.52)	<0.01
Had a sexually transmitted disease such as gonorrhoea, syphilis, chlamydia, genital warts, or genital herpes	1.10	(.44-2.76)	0.51
Had given money, gifts or drugs to exchange for sex	0.80	(.32-2.02)	0.41
Had sex with someone to get money, gifts or drugs	0.64	(.27-1.50)	0.21
Had injected street drugs, steroids or vitamins with a needle	.43	(.14-1.27)	0.10

Statistically significant at the p≤0.05
